# Cerebrospinal Fluid Dynamics Analysis Using Time-Spatial Labeling Inversion Pulse (Time-SLIP) Magnetic Resonance Imaging in Mice

**DOI:** 10.3390/jcm13154550

**Published:** 2024-08-04

**Authors:** Yusuke Tomita, Mitsuru Yagi, Fumiko Seki, Yuji Komaki, Morio Matsumoto, Masaya Nakamura

**Affiliations:** 1 Department of Orthopedic Surgery, Keio University School of Medicine, Tokyo 160-8582, Japan; mailyuto0531@yahoo.co.jp (Y.T.);; 2 Department of Orthopedic Surgery, School of Medicine, International University of Health and Welfare, Chiba 286-8520, Japan; 3 Department of Physiology, Keio University School of Medicine, Tokyo 160-8582, Japan; 4 Bioimaging Center, Central Institute for Experimental Medicine and Life Science, Kawasaki 210-0821, Kanagawa, Japan

**Keywords:** cerebrospinal fluid, live imaging, animal experiment

## Abstract

**Background/Objectives:** Abnormalities in cerebrospinal fluid (CSF) dynamics cause diverse conditions, such as hydrocephalus, but the underlying mechanism is still unknown. Methods to study CSF dynamics in small animals have not been established due to the lack of an evaluation system. Therefore, the purpose of this research study is to establish the time-spatial labeling inversion pulse (Time-SLIP) MRI technique for the evaluation of CSF dynamics in mice. **Methods:** We performed the Time-SLIP technique on 10 wild-type mice and 20 Tiptoe-walking Yoshimura (TWY) mice, a mouse model of ossification of the posterior longitudinal ligament (OPLL). We defined the stir distance as the distance of CSF stirring and calculated the mean ± standard deviation. The intraclass correlation coefficient of intraobserver reliability was also calculated. Furthermore, in TWY mice, the correlation coefficient between stir distance and canal stenosis ratio (CSR) was calculated. **Results:** The stir distance was significantly lower in TWY mice at 12 weeks and 17 weeks of age (1.20 ± 0.16, 1.21 ± 0.06, and 1.21 ± 0.15 mm at 12 weeks and 1.32 ± 0.21, 1.28 ± 0.23, and 1.38 ± 0.31 mm at 17 weeks for examiners A, B, and C). The intrarater reliability of the three examiners was excellent (>0.90) and there was a strongly negative correlation between stir distance and CSR in TWY mice (>−0.80). **Conclusions:** In this study, we established the Time-SLIP technique in experimental mice. This technique allows for a better understanding of CSF dynamics in small laboratory animals.

## 1. Introduction

Cerebrospinal fluid (CSF) is a clear, colorless fluid that serves vital functions in protecting and nourishing the brain and spinal cord [1]. It is produced in specialized structures called the choroid plexus located in the brain’s ventricles. From there, it follows a specific pathway, flowing through the ventricles and into the subarachnoid space, which surrounds the brain and spinal cord. CSF provides mechanical support, delivers nutrients, removes waste products, and acts as a cushion against impacts. The dynamics of CSF are regulated by its production, circulation, and absorption processes. CSF is continually produced and reabsorbed to maintain a stable volume and pressure. Its reabsorption primarily occurs through arachnoid granulations, which allow the CSF to be absorbed back into the bloodstream. The circulation of CSF is driven by arterial pulsations, movements of the brain and spinal cord, and the production–absorption balance [2]. Disruptions in CSF dynamics can lead to various conditions. Obstructions or impaired absorption can result in increased pressure within the brain, known as hydrocephalus. Conversely, decreased production or impaired circulation and absorption can lead to reduced CSF volume, affecting brain protection and nutrient delivery.

Our understanding of CSF dynamics, initially described by Cushing et al. in the early 20th century, has undergone significant transformation in recent years [3]. These dynamics, driven by motile cilia, heartbeat, respiration, and body movements, play a pivotal role in the proper functioning of the central nervous system (CNS) and organogenesis [4]. The relationship with respiration is particularly significant as it directly influences the pulsatile nature of CSF flow. Moreover, the glymphatic system, which facilitates waste clearance from the brain, relies heavily on the efficient movement of CSF.

Recent studies suggest that the choroid plexus may not be the sole or even the major source of CSF within the primate ventricular system, highlighting the importance of understanding brain barriers in fluid movement and the potential role of the glymphatic system [5]. Disturbances in CSF dynamics have been associated with a range of pathological conditions such as hydrocephalus in humans (and animal models) and spinal abnormalities (e.g., scoliosis) in fish [6,7]. Yet, despite our increasing knowledge of the importance of CSF flow, reliably imaging these oscillatory dynamics in small laboratory animals presents substantial technical challenges. Non-invasive techniques for measuring cerebrospinal fluid (CSF) dynamics include phase-contrast magnetic resonance imaging (PC-MRI), which measures CSF flow velocity by synchronizing data acquisition with the cardiac cycle, and cine magnetic resonance imaging (Cine MRI), which visualizes CSF movement over several cardiac cycles. Magnetic Resonance Spectroscopy (MRS) provides chemical composition information of CSF, while Diffusion Tensor Imaging (DTI) measures water molecule diffusion to infer CSF flow direction. Arterial Spin Labeling (ASL) uses magnetically labeled blood to measure cerebral blood flow, indirectly providing insights into CSF flow. Fourier Velocity Encoding (FVE) encodes velocity information into the MRI signal to measure CSF flow velocity, and oxygen-17 magnetic resonance imaging (17O-MRI) uses oxygen-17 as a tracer to study CSF dynamics. Each technique offers unique strengths and limitations, making them suitable for different aspects of CSF research [5]. Compared to other modalities, the Time-SLIP technique provides several advantages: it is non-invasive, does not require external contrast agents, offers high sensitivity, and enables longer visualization times. These attributes make it particularly suitable for repeated studies and critical for early diagnosis and research.

The time-spatial labeling inversion pulse (Time-SLIP) MRI technique is an advanced, non-invasive imaging method used to visualize cerebrospinal fluid (CSF) dynamics. Unlike conventional MRI techniques that require contrast agents, Time-SLIP uses the CSF itself as an endogenous tracer, reducing potential side effects. This technique involves using inversion pulses to label a specific volume of CSF, effectively “tagging” it. After the inversion pulse, a delay period allows the tagged CSF to move, and MRI scans are then acquired to visualize this movement, showing how CSF flows through the brain and spinal cord. The Time-SLIP technique is particularly useful for studying the oscillatory and pulsatile nature of CSF flow influenced by heartbeat, respiration, and body movements, leading to the realization that CSF, previously thought to flow unidirectionally, actually stirs [8]. Time-SLIP MRI has applications in investigating neurological conditions like hydrocephalus and spinal cord disorders and has been successfully applied to small animal models. This method offers dynamic, real-time visualization of CSF movement, providing detailed information about CSF flow dynamics with high sensitivity. Additionally, Time-SLIP allows for a longer visualization time compared to phase-contrast techniques, enhancing its utility in studying CSF dynamics.

In this study, our purpose is to apply the Time-SLIP technique to assess CSF dynamics in laboratory mice. We aim to verify its reliability and feasibility in this context, potentially providing a new avenue for studying CSF dynamics in small animals. There is limited established knowledge specifically on the cerebrospinal fluid (CSF) dynamics of Tiptoe-walking Yoshimura (twy/twy) mice, a mouse model of ossification of the posterior longitudinal ligament (OPLL), which is likely to have obstructed CSF flow [9,10]. OPLL can cause spinal cord compression and subsequent CSF flow obstruction, as demonstrated in various studies [11]. Given their genetic predisposition to spinal abnormalities, we hypothesized that twy/twy mice might exhibit altered CSF flow, providing insights into the impact of spinal deformities on CSF dynamics. By comparing the CSF dynamics of twy/twy mice to those of wild-type mice, we hope to further elucidate the effects of spinal abnormalities on CSF flow.

## 2. Materials and Methods

### 2.1. Experimental Animal Models

We aim to verify the reliability and feasibility of the Time-SLIP technique in this context, potentially providing a new avenue for studying CSF dynamics in small animals. Additionally, we employ the Time-SLIP technique on Tiptoe-walking Yoshimura (twy/twy) mice, a mouse model of ossification of the posterior longitudinal ligament (OPLL), which is likely to have obstructed CSF flow. By comparing the CSF dynamics of twy/twy mice to those of wild-type mice, we hope to further elucidate the effects of spinal abnormalities on CSF flow. The number of mice chosen for this study was determined based on a power calculation to ensure statistical significance, balanced with the availability of mice and the funding and support received (Supplemental Data S1).

To achieve this, 10 wild-type B57/BL6 mice (5 mice at 12 weeks old and 5 mice at 17 weeks old) were obtained from Clea Japan INC (Meguro, Tokyo, Japan), and 20 twy/twy mice (10 mice at 12 weeks old and 10 mice at 17 weeks old) were obtained from the Central Institute for Experimental Medicine and Life Science (Kawasaki, Japan). These mice were housed under a 12 h/12 h light/dark cycle with free access to food and water. This study follows the recommendations in the ARRIVE guidelines.

### 2.2. Image Acquisition

We conducted 7T MRI (BioSpec 70/16; Bruker BioSpin, Ettlingen, Germany) on mice under isoflurane anesthesia (ISOFLURANE Inhalation Solution, Viatris Inc., Pittsburgh, PA, USA). The mice were induced into an anesthetic state with 3–4% isoflurane and maintained at 1–2% isoflurane during the MRI measurements. Throughout the MRI procedure, the mice were continuously monitored for respiratory rate and body temperature, and they were provided with adequate heating via a warm water pad to maintain their physiological condition. We acquired anatomical midsagittal section images of the brain using a rapid acquisition with Relaxation Enhancement (RARE) sequence with the following parameters: effective echo time (eTE) = 40 ms, repetition time (TR) = 2000 ms, RARE factor = 8, number of averages = 1, spatial resolution = 78 × 78 × 1000 (μm)^3^, and number of slices = 1.

We combined the Time-SLIP method to visualize the CSF flow with Inversion Recovery (IR) pulses and true-FISP acquisition and imaged with the following parameters: TE = 2.4 ms, TR = 4.8 ms, flip angle = 60 deg., inversion time = 3000 ms, spatial resolution = 78 × 78 × 1000 (μm)^3^, and number of slices = 1. We acquired two images: one with the labeling pulse set to the fourth ventricle and one with the labeling pulse set outside the brain region. Labeling pulses are required in the Time-SLIP technique to selectively invert the magnetization of a specific volume of CSF, effectively “tagging” it. This process enables the visualization of the movement of the tagged CSF over time. The delay period after the inversion pulse allows the tagged CSF to flow, and subsequent MRI scans capture this movement, providing dynamic images of CSF flow. This technique is crucial for studying CSF dynamics as it highlights the flow patterns and its interactions with various physiological factors like heartbeat and respiration (Supplemental Data S2).

**Figure 1:**
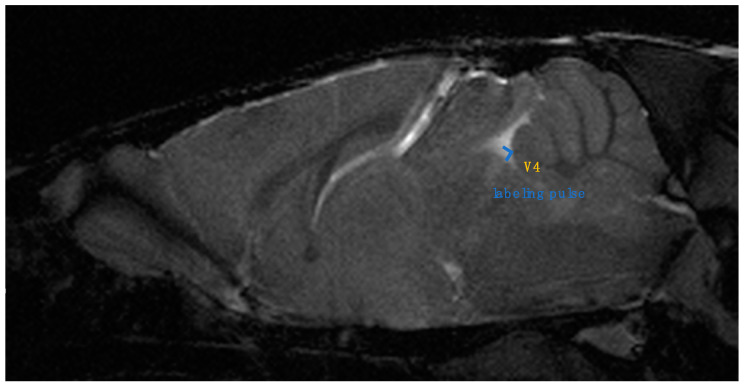
Sagittal slice of in vivo MRI mouse brain image. Labeling pulse for fourth ventricle (V4) was placed in section perpendicular to midsagittal section.

To account for magnetization transfer (MT) effects, the pulse was placed outside the brain region rather than without the labeling pulse. In the case of the labeling pulse used outside the brain region, the specific placement was decided carefully to ensure that it did not intersect with any region where CSF flow could be expected. The pulse was positioned in an area that was sufficiently far from the brain and the spinal cord to avoid any accidental labeling of the CSF. To guarantee this, the labeling pulse was placed at an area above the cranium, in the air, where we could be confident that no CSF flow is present. The labeling pulse for the fourth ventricle was placed in a section perpendicular to the midsagittal section (Figure 1). The rostral–caudal angle was defined as a line perpendicular from the dorsal apex of the fourth ventricle to the ventral side. The thickness of the labeling pulse was kept constant at 0.3 mm. We saved all image data as DICOM (Digital Imaging and Communications in Medicine) files and exported them to OsiriX (Pixmeo, Geneva, Switzerland) for analysis.

### 2.3. Examiners and Measurements

**Figure 2:**
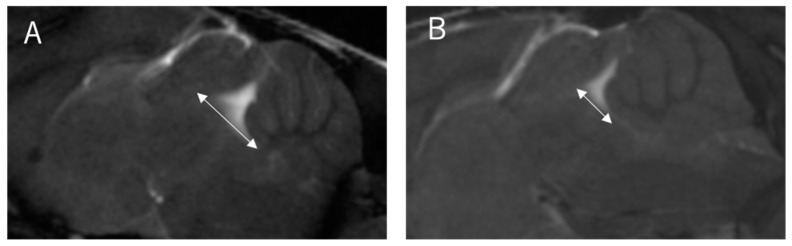
The distance of CSF stirring in the sagittal plane within a single time period at the bottom of the fourth ventricle. Stir distance (two-headed arrow shows stir distance). (**A**) Stir distance in 12-week-old wild-type mouse, (**B**) stir distance in 12-week-old twy/twy mouse.

**Figure 3:**
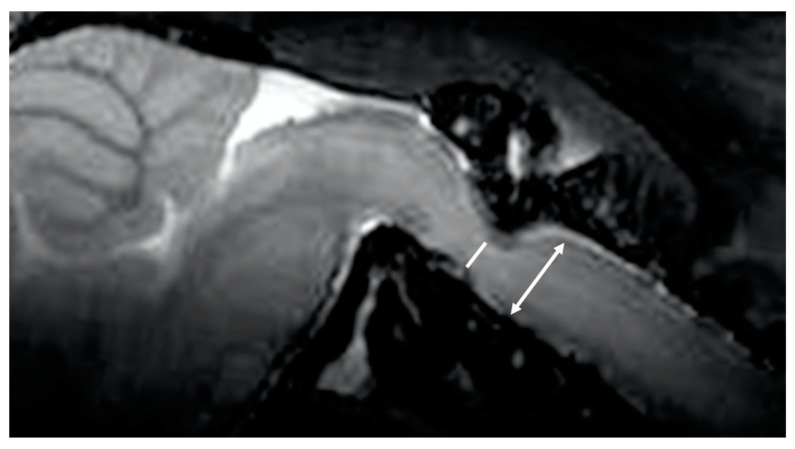
Canal stenosis ratio. Canal stenosis ratio = 100 − (antero-posterior diameter of spinal cord) [White line]/antero-posterior diameter of spinal canal [two-headed arrow] × 100.

Two board-certified physicians and one senior author who specialized in the imaging of the CNS in laboratory animals independently measured the images of 30 mice (10 wild-type and 20 twy/twy mice) taken with the Time-SLIP technique 3 times. The examiners were blinded to the genotype of the mice. We defined the stir distance as the distance of CSF stirring in the sagittal plane within a single time period (3 s) in the fourth ventricle, where there is relatively more space, and three examiners took measurements 3 times for each mouse (Figure 2). Furthermore, the examiners also measured the antero-posterior diameter of the spinal cord and spinal canal on the MRI sagittal plane and calculated the canal stenosis ratio (CSR). (Figure 3) We examined the correlation between stir distance and CSR using Pearson’s correlation coefficient. Additionally, to eliminate the potential impact of respiration on the dynamics of CSF, the respiratory rates of wild-type and twy/twy mice were recorded and compared.

### 2.4. Statistical Analysis

We calculated the mean ± standard deviation (SD) of the stir distance obtained from twy/twy mice and wild-type mice using the data from the 3 examiners. We performed a *t*-test for the stir distance for each age group (12- and 17-week-old mice) and combined ages (12 + 17-week-old mice). The level of significance was set at *p* < 0.05. The intraclass correlation coefficient (ICC) of the intraobserver reliability of measurements was also calculated. We classified the ICC values according to the criteria introduced by Aubin et al.; <0.24, 0.25–0.49, 0.50–0.69, 0.70–0.89, and 0.90–1.0 were considered to be poor, low, fair to moderate, good, and good to excellent, respectively [12].

Additionally, we performed correlation analysis to examine the relationship between stir distance and canal stenosis ratio (CSR) in twy/twy mice. The correlation coefficient (r) and the corresponding *p*-value were reported to assess the strength and significance of this relationship. All statistical analyses were performed using SPSS ver. 25.0 (IBM Corp., Armonk, NY, USA).

### 2.5. Histological Analysis

The twy/twy mice were anesthetized and transcardially perfused with 4% paraformaldehyde (PFA) in 0.1 M PBS, and the cervical spine was removed. They were fixed with 4% PFA and immersed in 10% formalin formate for 3 days for decalcification. After decalcification, a paraffin block section with a thickness of 5 μm on the sagittal plane was made, and hematoxylin and eosin (HE) staining was performed. The samples were observed with a microscope (BZ9000; Keyence Co., Osaka, Japan).

## 3. Results

### 3.1. Stir Distance Measured by the Time-SLIP Technique in Wild-Type Mice

The measured stir distance values in 12-week-old wild-type mice by examiners A, B, and C were 1.80 ± 0.13 mm^3^/s, 1.76 ± 0.12 mm^3^/s, and 1.69 ± 0.20 mm^3^/s, respectively. In the 17-week-old cohort, these measurements were 1.57 ± 0.12 mm^3^/s, 1.56 ± 0.09 mm^3^/s, and 1.68 ± 0.09 mm^3^/s, respectively (Table 1). The stir distance values measured by the three examiners were 1.75 ± 0.16 mm^3^/s for 12-week-old and 1.60 ± 0.11 mm^3^/s for 17-week-old wild-type mice.

### 3.2. Respiratory Rates of Wild-Type and Twy/Twy Mice

The respiratory rates, expressed in breaths per minute (bpm), were found to be 43.9 ± 7.9 bpm for wild-type mice and 38.9 ± 8.1 bpm for twy/twy mice. The difference between these rates was analyzed statistically, yielding a *p*-value of 0.17. This result suggested that the difference in respiratory rates between the two types of mice is not statistically significant. Therefore, any observed variations in CSF dynamics between wild-type and twy/twy mice cannot be attributed to differences in their respiratory rates. These findings further strengthen our study by ruling out respiration as a confounding factor in the analysis of CSF dynamics.

### 3.3. Comparisons of Star Distance between Wild-Type Mice and Twy/Twy Mice

**Figure 4:**
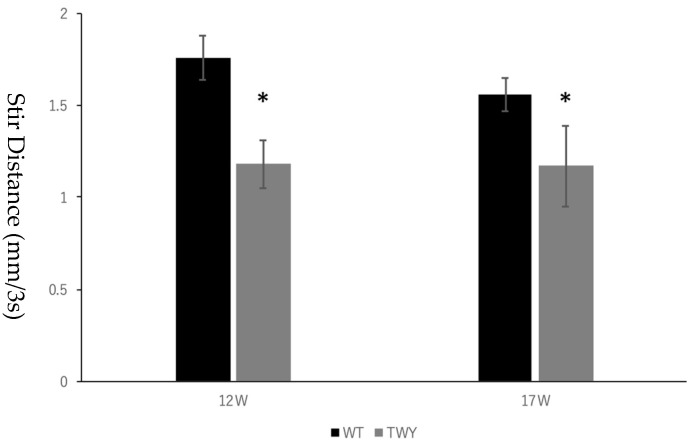
Comparing the stir distance of wild-type mice and twy/twy mice at 12 weeks and 17 weeks, it was significantly lower in twy/twy mice when measurements were averaged from three examiners. * indicates statistically significant.

The stir distance was 1.18 ± 0.17 mm^3^/s, 1.18 ± 0.13 mm^3^/s, and 1.20 ± 0.16 mm^3^/s for 12-week-old twy/twy mice and 1.18 ± 0.23 mm^3^/s, 1.17 ± 0.22 mm^3^/s and 1.18 ± 0.33 mm^3^/s for 17-week-old twy/twy mice for examiners A, B, and C, respectively (Table 1). Comparing the stir distance of wild-type mice with that of twy/twy mice at 12 weeks of age, it was significantly lower in twy/twy mice (*p* < 10^−14^, 10^−18^, and 10^−10^ for examiners, A, B, and C, respectively). Similarly, when comparing the stir distance of wild-type mice with that of twy/twy mice at 17 weeks of age, it was significantly lower in twy/twy mice (*p* < 10^−6^, 10^−7^, and 10^−6^ for examiners A, B, and C, respectively). The stir distance values measured by the three examiners were 1.19 ± 0.15 mm^3^/s for 12-week-old and 1.17 ± 0.26 mm^3^/s for 17-week-old twy/twy mice. In the same way, the stir distance of twy/twy mice was significantly lower when measurements were averaged from the three examiners (*p* < 10^−40^ for 12-week-old and 10^−18^ for 17-week-old mice) (Figure 4).

### 3.4. Reliability of Stir Distance Measurements in Wild-Type Mice

The intrarater reliability of the three examiners was excellent, with mean measurements of 0.990 (0.979–0.996), 0.990 (0.980–0.996), and 0.966 (0.929–0.985) for examiners A, B, and C, respectively. (Table 1) Similarly, the inter-rater reliability was excellent, with a mean measurement of 0.990 (0.980–0.996).

### 3.5. Correlation between Stir Distance and CSR

**Figure 5:**
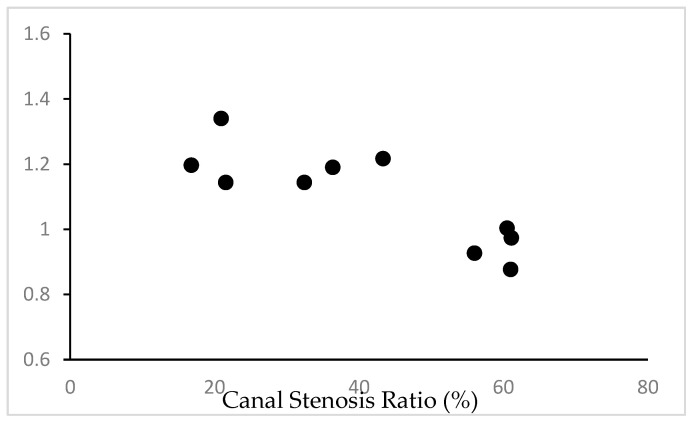
Examining correlation between stir distance and CSR in 10 TWY mice. There was a strong negative correlation. (The result of examiner B, with the highest CC.)

The correlation coefficient between stir distance and CSR was −0.80, −0.84, and −0.83 for examiners A, B, and C, respectively, with *p*-values all <0.05 (*p* = 0.02, 0.01, 0.02), indicating a statistically significant negative correlation. The result of examiner B, with the highest correlation coefficient, clearly shows that there was a strong negative correlation between stir distance and CSR in TWY mice (Figure 5).

### 3.6. Histological Assessment

**Figure 6:**
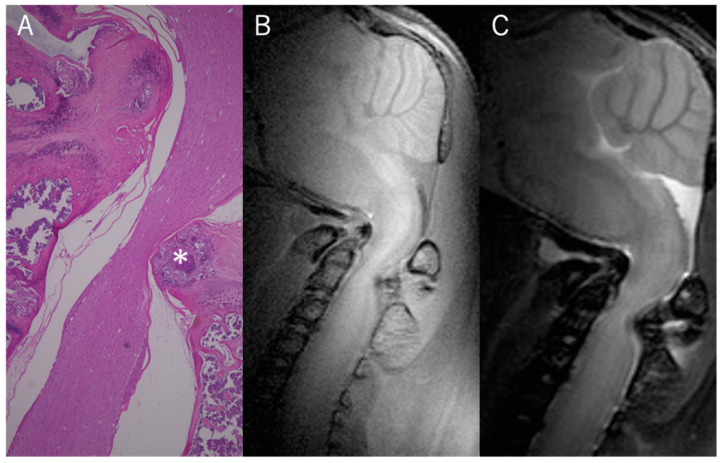
Histological evidence of significant ossification and compression of the spinal cord in twy/twy mice (*). (**A**) Microphotographs of hematoxylin and eosin (H&E)-stained sagittal sections of the cervical spine of 17-week-old twy/twy mice. (**B**) T1WI sagittal MRI showed obvious spinal cord compression resulting from ectopic calcification. (**C**) T2WI sagittal MRI showed obvious spinal cord compression resulting from ectopic calcification.

The histopathological examination of the spinal canal in craniovertebral lesions of 17-week-old twy/twy mice showed significant ossification and resulting compression of the spinal cord, which strongly suggested the presence of decreased CSF flow (Figure 6).

## 4. Discussion

### 4.1. Historical Context of CSF Circulation

The concept of CSF circulation is based on a hypothesis proposed in the early 20th century by Harvey Cushing, a neurosurgeon in the U.S., as the third circulation following blood and lymph circulation, and published by Weed et al., who worked with Cushing. In other words, CSF is produced in the choroid plexus in the ventricles, flows from the lateral ventricles and third ventricle through the mesencephalic aqueduct to the fourth ventricle, and then ascends through the foramen of Luschka and Magendie to the basilar ventricle, where it is absorbed from the arachnoid granules on the parietal surface of the brain into the sinus of the superior arrowhead and returns to the body circulation [3,13]. However, Yamada et al. established the Time-SLIP technique [8] to visualize CSF dynamics using spinal fluid itself as an endogenous tracer and clarified that CSF, which was previously thought to flow unidirectionally due to the pulsation of the choroid plexus, is stirred.

### 4.2. Conventional Techniques and Limitations

Radioisotope (RI) scintigraphy and metrizamide CT cerebral alveolar angiography have been the conventional techniques to observe CSF circulation. However, these techniques cannot observe CSF under physiological conditions because the intracranial environment is altered by injecting contrast media. In addition, the mass and viscosity of tracers such as RI tracers and metrizamide are different from those of the actual CSF, so they do not accurately represent the dynamics of the CSF.

Since the late 1980s, phase-contrast (PC) cine MRI has been used to capture the pulsation of the CSF non-invasively [14]. In this technique, the heartbeat and CSF pulsations are synchronized, and the data are acquired and averaged. Therefore, the CSF flow is observed within a single heartbeat, or one second. However, spinal fluid pulsates not only with the heartbeat but also with respiration, a fact that is observed by physicians during surgery. Therefore, it is questionable whether PC cine MRI, which collects data using only the heartbeat as the driving force of CSF, shows physiological CSF dynamics in vivo.

In addition to the aforementioned techniques, MRI analysis using gadolinium-based contrast agents (GBCAs) and 17O-labeled water as a tracer can be used to analyze CSF dynamics in mice. However, these techniques require the injection of an external tracer [15]. Recently, two-photon microscopy has also been reported, but this technique has spatial limitations of the region of interest. The Time-SLIP technique solves these problems [16,17]. There is no report on the application of the Time-SLIP technique to analyze CSF dynamics in mice. The present study first described the utility and reliability of the Time-SLIP technique to evaluate CSF dynamics in laboratory animals such as mice.

### 4.3. Factors Influencing CSF Dynamics

The dynamics of CSF have traditionally been understood to be largely influenced by factors such as heartbeat, motile cilia, and body movements. More recently, the significance of respiratory-driven CSF dynamics has been increasingly recognized. During surgeries, for instance, it is common to observe respiratory-induced pulsation of the dura mater, which can substantially contribute to CSF movement. Consequently, a more comprehensive portrayal of CSF dynamics may be achieved by considering both cardiac and respiratory-driven CSF oscillations. In addition to the aforementioned techniques, MRI analysis using gadolinium-based contrast agents (GBCAs) and 17O-labeled water as a tracer can be used to analyze CSF dynamics in mice. However, these techniques require the injection of an external tracer [16]. Recently, two-photon microscopy has also been reported, but this technique has spatial limitations of the region of interest. The Time-SLIP technique solves these problems [16,17]. There is no report on the application of the Time-SLIP technique to analyze CSF dynamics in mice. The present study first described the utility and reliability of the Time-SLIP technique to evaluate CSF dynamics in laboratory animals such as mice.

### 4.4. Time-SLIP MRI and the Stir Distance Parameter

In this study, we employed the ‘stir distance’ parameter, defined as the distance of CSF stirring in the sagittal plane within a single time period, to evaluate CSF dynamics in both wild-type mice and Tiptoe twy/twy mice. We have often observed respiratory pulsation of the dura during surgery for spinal disorders, particularly when the pressure on the dura is released by decompression. It is conceivable that this respiratory pulsation acts as a primary driver of CSF movement. Hence, the CSF dynamics in twy/twy mice, which exhibit spinal canal narrowing, may present notable differences compared to wild-type mice. Indeed, our results indicate a significantly lower stir distance in twy/twy mice compared to wild-type mice at both 12 and 17 weeks. Specifically, the stir distance in twy/twy mice was reduced to 65–85% of the value observed in wild-type mice. We believe the values obtained for the CSF dynamics in wild-type mice in this study can provide a valuable reference for future investigations into various pathological conditions related to CSF dynamics. Additionally, we found a strong negative correlation between stir distance and CSR. Applying the Time-SLIP technique, our study challenges the traditional understanding of unidirectional CSF flow. Our study challenges this traditional view by employing a novel parameter, ‘stir distance’, and the time-spatial labeling inversion pulse (Time-SLIP) technique. Our results, based on studies of wild-type mice and Tiptoe twy/twy mice, suggest a complex, possibly oscillatory, flow pattern within the ventricular system, countering the notion of unidirectional CSF flow. However, to accurately depict CSF dynamics, it is essential to consider factors like heartbeat, respiration, and body movements. We note the pulsations of the CSF due to cardiac and respiratory cycles as significant contributors to the CSF dynamics. This observation is supported by our surgical experiences, where the respiratory-induced pulsation of the dura mater was often noticeable. Despite our findings and observations, we acknowledge that the complexity of CSF dynamics cannot be fully represented using a single parameter like ‘stir distance’ or a single-shot imaging technique like Time-SLIP MRI. These methods provide valuable insights but may oversimplify the intricate, pulsatile nature of CSF motion.

### 4.5. Study Limitations and Future Directions

Although our study sheds new light on the application of the Time-SLIP MRI technique for the study of CSF dynamics in small animals, certain limitations should be noted. First, our approach employs a single-shot imaging technique that does not fully account for the pulsatile nature of CSF flow driven by respiration and heartbeat. This might prevent a comprehensive understanding of CSF dynamics, which are intrinsically influenced by these physiological pulsations. Secondly, our study utilized the ‘stir distance’ metric, defined as the distance traversed by CSF within a single time period. The choice to use the fourth ventricle as the representative region for measuring CSF dynamics has been met with some skepticism, given its relatively minor contribution to total CSF volume. We acknowledged this potential confounder and conducted a comparative analysis of fourth ventricle volumes in wild-type and TWY mice. The results did not reveal a significant difference (0.553 ± 0.126 mm^3^ vs. 0.540 ± 0.111 mm^3^, *p* = 0.79), thus indicating that variations in fourth ventricle volume did not significantly influence our findings.

In studies involving humans and larger animals, the pulsatile motion of CSF has been successfully visualized using cine phase-contrast MRI, which yields a time-resolved velocity map of CSF flow. However, the applicability of this technique is limited in our context, due to the relatively small size and rapid heart rate of mice. As such, the resolution necessary for an effective implementation of this method is currently out of reach. While the use of a single-shot imaging technique provides a snapshot of CSF dynamics, it may oversimplify the complex and cyclic nature of CSF motion. Therefore, future studies could consider incorporating time-resolved imaging techniques or using mathematical models to simulate CSF pulsatility and better understand the complex interplay between CSF and cardiovascular rhythms. Additionally, we were unable to automate the measurements of the images in this study because developing the necessary software is beyond the current study’s scope and will be addressed in future research. Consequently, there is a possibility of error or bias through the manual measurement of values. However, the excellent intra- and interobserver reliability of the Time-SLIP technique demonstrates the usefulness of this method.

Furthermore, the skepticism towards the use of the fourth ventricle region stems from its relatively small contribution to the overall CSF volume and the potential for localized dynamics not representative of the entire CSF system. One can argue that this could limit the generalizability of our findings to broader CSF dynamics. Additionally, other regions may provide more comprehensive insights into CSF flow and dynamics, raising doubts about the sole reliance on the fourth ventricle region. Despite this, our comparative analysis indicated no significant differences in fourth ventricle volumes between wild-type and TWY mice, suggesting that our measurements were not significantly biased by this choice.

While our study has demonstrated that the Time-SLIP technique can provide valuable information about CSF dynamics in small animals, it also highlights the need for further developments in imaging techniques to comprehensively assess CSF dynamics, particularly in light of its pulsatile nature. With ongoing advancements in imaging technology, we are hopeful that these limitations can be overcome in future studies.

This study represents a crucial step forward in applying the Time-SLIP MRI technique to study CSF dynamics in small animals like mice, but several avenues for future research have been identified. One significant direction could involve expanding our study’s methodology to include the use of multiple interval times in the Time-SLIP technique. Our choice of a single interval time may not fully capture the complex flow patterns of the CSF, potentially affecting the accuracy of our results. Future studies should consider using multiple interval times to create a more comprehensive depiction of CSF dynamics. In addition to this, given the significant ossification and compression of the spinal cord observed in the TWY mice, it may be interesting to examine CSF dynamics in the CSF space at the spinal canal stenosis lesion using the Time-SLIP method. This could provide a useful way to study the impact of spinal canal stenosis on CSF dynamics, thereby advancing our understanding of this condition and offering potential pathways for its treatment. Finally, the pulsatile nature of CSF flow—linked to heartbeat and respiration—should be incorporated in future studies for a more holistic understanding of CSF dynamics. This could potentially involve the development or adaptation of methods that can capture this pulsatile motion, which would provide a more accurate representation of in vivo CSF dynamics. Our study has provided a solid foundation for such future explorations, and we look forward to seeing these possibilities come to fruition in the field of small animal CSF dynamics research.

## 5. Conclusions

In our study, we aimed to apply the Time-SLIP technique to assess CSF dynamics in small laboratory animals, specifically using wild-type and Tiptoe twy/twy mice. Our hypothesis was that the Time-SLIP MRI would detect abnormalities in CSF dynamics in these experimental models. The findings of our study confirmed this hypothesis by demonstrating a significantly lower stir distance in twy/twy mice compared to wild-type mice, indicating altered CSF flow due to spinal canal narrowing. The utility of the Time-SLIP technique was successfully demonstrated, showing its potential to enhance our understanding of CSF dynamics and aid in the research of related pathological conditions. This non-invasive method provides a valuable tool for visualizing and measuring CSF movement in small animals without the need for external tracers.

Future research can build on the current findings by expanding the use of multiple interval times in the Time-SLIP technique to capture more complex CSF flow patterns. Additionally, advancements in imaging technology, such as higher resolution and faster acquisition times, are required to fully capture the pulsatile nature of CSF dynamics influenced by heartbeat and respiration. By addressing these aspects, future studies can provide a more comprehensive assessment of CSF dynamics and further our understanding of various pathological conditions related to CSF flow.

We look forward to these advancements, which will allow for more detailed and accurate investigations into CSF dynamics, ultimately contributing to better diagnosis and treatment of related neurological disorders.

## Figures and Tables

**Table 1:** Three examiners (A–C) measured the stir distance of the image taken using the Time-SLIP technique 3 times. We calculated the mean ± standard deviation (SD) (mm^3^/s) and intraobserver reliability of stir distance measurements.

	Examiner A	Examiner B	Examiner C
WT 12 w (*n* = 5)	1.80 ± 0.13	1.76 ± 0.12	1.69 ± 0.20
WT 17 w (*n* = 5)	1.57 ± 0.12	1.56 ± 0.09	1.68 ± 0.09
TWY 12 w (*n* = 10)	1.18 ± 0.17	1.18 ± 0.13	1.20 ± 0.16
TWY 17 w (*n* = 10)	1.18 ± 0.23	1.17 ± 0.22	1.18 ± 0.33
ICC (95% CI)	0.990 (0.979–0.996)	0.990 (0.980–0.996)	0.966 (0.929–0.985)

## Data Availability

The datasets generated during and/or analyzed during the current study are not publicly available but are available from the corresponding author on reasonable request.

## Supplementary Materials

The following supporting information can be downloaded at: https://www.mdpi.com/article/10.3390/jcm13154550/s1, Supplemental Data S1: Detailed description of statistical analyses; Supplemental Data S2: Detailed description of Time-SLIP MRI.
