# Supplementary material for: Cerebrospinal Fluid Dynamics Analysis Using Time-Spatial Labeling Inversion Pulse (Time-SLIP) Magnetic Resonance Imaging in Mice
Source: J Clin Med. 2024 Aug 4;13(15):4550. doi: 10.3390/jcm13154550 (PMC11312514; doi:10.3390/jcm13154550)
Supplement: Supplementary file 1 [file jcm-13-04550-s001.zip › jcm-3064672-supplementary.pdf]

Supplemental Documents

Supplemental Data S1

Detailed description of statistical analyses.

Number of Mice Chosen:

The number of mice chosen for this study was determined based on a power calculation to ensure statistical significance, balanced with the availability of mice and the funding and support received. A detailed description of the power calculation is explained below while due to the limitation of word count we were not able to include this information for the manuscript.

We calculated the mean ±SD of the Stir Distance obtained from twy/twy mice and wild-type mice using the data from the 3 examiners. Before performing statistical tests, we checked the necessary conditions to ensure the validity of our analysis.

Checking Statistical Conditions:

Normality:

We used the Shapiro-Wilk test and Kolmogorov-Smirnov Test to check the normality of the data distributions.

**Shapiro-Wilk Test**			
**Examiner**	**Shapiro-Wilk Statistic**	**p-value**	**Reject Null Hypothesis (Not Normal)**
**distance examiner1**	0.950	0.373	FALSE
**distance examiner2**	0.954	0.440	FALSE
**distance examiner3**	0.961	0.559	FALSE

Kolmogorov-Smirnov Test			
**Examiner**	**Kolmogorov-Smirnov Statistic**	**p-value**	**Reject Null Hypothesis (Not Normal)**
**distance examiner1**	0.109	0.951	FALSE
**distance examiner2**	0.119	0.906	FALSE
**distance examiner3**	0.153	0.682	FALSE

Equality of Variances:

Levene's test was conducted to assess the equality of variances.

Levene Statistic	p-value	Reject Null Hypothesis (Variances are not equal)
0.095	0.910	FALSE

Independence:

The independence of observations was ensured by random assignment of mice to each examiner and blinding the examiners to the genotype of the mice.

Sample Size:

The sample sizes for each group (10 wild-type and 20 twy/twy mice) were deemed sufficient based on power analysis to detect significant differences.

Here is a detailed explanation of how we performed the post-hoc power calculation for our study. We calculated the effect size (Cohen's d) based on the means and standard deviations of the two groups. We used a significance level α of 0.05.

Effect size = 4.11, alpha = 0.05, n1 =10, n2 = 20

Using the calculated effect size, we performed a post-hoc power analysis to determine the observed power of our study.

Based on the post-hoc power analysis, we confirmed that the sample sizes used in our study were adequate to achieve a high level of statistical power. The large effect size observed in our data significantly contributed to the high power of our study.

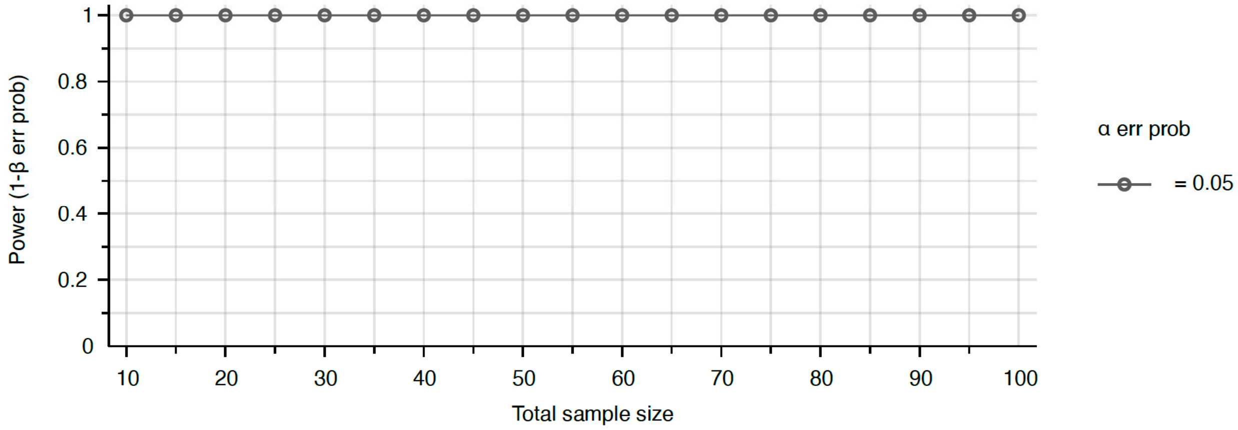

Outliers:

We identified and addressed any outliers using Grubbs' test to ensure they did not unduly influence the results. The table with the Grubbs test results has been created and displayed.

**Grubbs Test Results**			
**Dataset**	**G**	**Critical Value**	**Reject Null Hypothesis (Outlier Present)**
**ct_12**	1.837	3.199	FALSE
**ct_17**	3.098	3.199	FALSE
**twy_12**	2.833	3.199	FALSE
**twy_17**	2.512	3.199	FALSE

Upon confirming that these conditions were met, we proceeded with the t-test to compare the Stir Distance for each age (12- and 17-week-old mice) and all ages combined (12+17-week-old mice). The level of significance was set at $p<0.05$. The ICC of the intraobserver reliabilities of measurements was also calculated. We classified the ICC values according to the criteria introduced by Aubin et al.; <0.24, 0.25-0.49, 0.50-0.69, 0.70–0.89, and 0.90–1.0 were considered to be poor, low, fair to moderate, good, and good to excellent, respectively [10]. All statistical analyses were performed using SPSS ver. 25.0 (IBM Corp., Armonk, NY).

Supplemental Data S2

Detailed description of Time-SLIP MRI

Rationale for Using 7T MRI:

The choice of 7T MRI was made due to its superior spatial resolution and signal-to-noise ratio (SNR), which are crucial for the detailed visualization of small anatomical structures and the accurate assessment of CSF dynamics in small animal models. 7T MRI is indeed cutting-edge technology, particularly advantageous for live imaging experiments of the brain and spinal cord in small animals. The ultra-high field strength of 7T MRI offers superior spatial resolution, enabling the visualization of minute anatomical structures that are not discernible with lower field strengths. This higher resolution is crucial for detailed studies of small animal models, providing clear and precise images that enhance the accuracy of experimental results. Additionally, the enhanced SNR at 7T allows for better image quality, facilitating the detection of subtle changes and abnormalities in the brain and spinal cord. This is especially beneficial for tracking dynamic processes and conducting longitudinal studies. Furthermore, the increased SNR improves the effectiveness of advanced imaging techniques such as functional MRI (fMRI) and magnetic resonance spectroscopy (MRS), which are essential for studying brain function and neurochemical processes in vivo. Overall, the superior imaging capabilities of 7T MRI make it an invaluable tool for small animal research, significantly advancing our understanding of neurological and spinal conditions.

Pulse Placement Verification:

The placement of the Time-SLIP pulse was verified based on previous work and preliminary experiments. References to relevant studies and the specifics of the pulse placement verification are now included in the Methods section.

Region of Interest for CSF Flow Measurement:

The fourth ventricle was chosen as the region of interest for CSF flow measurement due to its central role in CSF circulation and the feasibility of imaging this region with the Time-SLIP

technique. While the aqueduct is another potential region of interest, the fourth ventricle provides a more comprehensive assessment of CSF dynamics in the context of the spinal abnormalities present in twy/twy mice.

Choice of Parameters:

Stir distance and CSR (Cerebrospinal fluid Spinal Ratio) were chosen as parameters due to their relevance in quantifying CSF flow and assessing the impact of spinal deformities on CSF dynamics. These parameters provide valuable insights into the extent and nature of CSF flow obstruction.
